# The Effect of COVID-19 on Loneliness in the Elderly. An Empirical Comparison of Pre-and Peri-Pandemic Loneliness in Community-Dwelling Elderly

**DOI:** 10.3389/fpsyg.2020.585308

**Published:** 2020-09-30

**Authors:** Theresa Heidinger, Lukas Richter

**Affiliations:** ^1^Division of Gerontology and Health Research, Karl Landsteiner University of Health Sciences, Krems an der Donau, Austria; ^2^Institute for Sociology and Social Research, Vienna University of Economics and Business, Vienna, Austria

**Keywords:** loneliness, elderly, Covid-19, propensity score matching, health and well-being, comparative analysis

## Abstract

Old-age loneliness is a global problem with many members of the scientific community suspecting increased loneliness in the elderly population during COVID-19 and the associated safety measures. Although hypothesized, a direct comparison of loneliness before and during the pandemic is hard to achieve without a survey of loneliness prior to the pandemic. This study provides a direct comparison of reported loneliness before and during the pandemic using 1:1 propensity score matching (PSM) on a pre- and a peri-pandemic sample of elderly (60+ years) individuals from Lower Austria, a county of Austria (Europe). Differences on a loneliness index computed from the short De Jong Gierveld scale were found to be significant, evidencing that loneliness in the elderly population had in fact risen slightly during the COVID-19 pandemic and its associated safety measures. Although the reported loneliness remained rather low, this result illustrated the effect of the “new normal” under COVID-19. As loneliness is a risk factor for physical and mental illness, this result is important in planning the future handling of the pandemic, as safety measures seem to have a negative impact on loneliness. This work confirms the anticipated increase in loneliness in the elderly population during COVID-19.

## Introduction

With Covid-19 safety measures employed by governments across the globe, the impacts on the elderly population beyond morbidity and mortality have been heavily discussed in both scientific and political forums. Old-age loneliness, an important public health issue long before the outbreak (Victor et al., 2005), has been a primary concern, with members of the scientific community expecting loneliness to increase during the pandemic measures as lockdown and social distancing are instated for elderly citizens in the pretext of health safety (Armitage and Nellums, 2020; Ayalon et al., 2020; Brooke and Jackson, 2020). Loneliness has been associated with perceived stress and low social support (Cacioppo et al., 2006), which could be plausible effects of the Covid-19 measures. A subjective state, loneliness is defined as a discrepancy between desired and perceived quality and quantity of social relationships (Walton et al., 1991) and has been shown to be associated with poor mental and physical health (Cacioppo et al., 2006; Cohen-Mansfield et al., 2016) and to be a risk factor for of serious illness (Valtorta et al., 2016) and mortality (Steptoe et al., 2013; Holt-Lunstad et al., 2015). An increase in loneliness in response to COVID-19 measures may therefore have dangerous consequences, especially for the group of the already vulnerable elderly. Loneliness is hypothesized to increase with restrictions such as stay-at-home orders, which diminish elderly people’s in-person social encounters and thereby may be negatively affecting social connectedness (Lee et al., 2001), which has been shown to be related to in-person interactions (Sandstrom and Dunn, 2014). It is possible, however, that social interaction via alternative routes (phone calls, video chats) has helped compensate for these in-person contacts. In fact, there has been evidence that social connectedness has not been affected during the pandemic among adults (Folk et al., 2020). This could be similar in an older demographic, as it is plausible that virtual social contact with the elderly may have increased during the pandemic (possibly due to social reciprocity norms). Therefore, it remains unclear whether loneliness has in fact increased or decreased among the group of the elderly during this pandemic. This study empirically examines changes in loneliness in community-based elderly from Lower Austria, a county in Austria, comparing data from before and during the Covid-19 social distancing measures.

Austria was one of the first European countries to respond to the viral outbreak by implementing first protective measures at the end of February. A national shutdown was enforced by the government on March 10th with the government presenting a stay-at home order for all citizens on March 15th (BMSGPK, 2020), which was upheld until the end of April. Thereafter, citizens were asked to maintain a one-meter distance in public spaces, wear masks, and only meet in small groups of people. The data used in this study was collected before and during the lockdown of the country, enabling us to analyze changes in reported loneliness among elderly citizens.

## Materials and Methods

### Study Design and Sample Characteristics

Data of two surveys regarding the health and well-being of older people were used to create the study sample: a pre-pandemic survey held between April and July 2019 (*n* = 2042) and a peri-pandemic survey held between April and May 2020 (*n* = 521). Both surveys were standardized, representative community-based telephone surveys with elderly residents of Lower Austria (60+). In the large-scale pre-pandemic survey (duration ≈ 1 h), participants were asked about their current health status and health upkeep. In the smaller, peri-pandemic survey (duration ≈ 30 min) participants were asked about their current health status as well as about perceived behavioral changes due to the implemented social distancing measures. Loneliness and social support were surveyed in both cases. In both surveys, sampling was done based on municipality size using random sampling with age screening.

### Analysis

**FIGURE 1:**
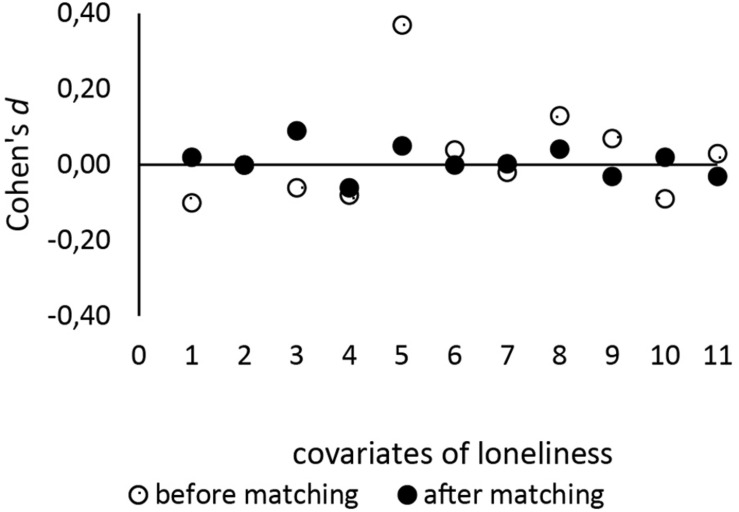
Standardized differences between survey time points before and after PSM. The figures demonstrate the contraction of the standard differences of covariates of loneliness before and after PS matching. Numbers on the *x*-axis denote the covariates: 1 = gender, 2 = marital status, 3 = number of living children, 4 = employment status, 5 = education level, 6 = number of people in the household, 7 = level of care allowance, 8 = income level, 9 = subjective health, 10 = age, 11 = social support.

IBM SPSS version 26 was used for analysis. A comparison between pre-pandemic versus peri-pandemic groups was done using an independent *t*-test to assess the significance of a possible difference in loneliness score. Statistical measures were interpreted as per scientific standard. In order to achieve a viable comparison of reported loneliness between the pre- and peri-pandemic measurement, propensity score matching (PSM) was performed using the “psmatching” program developed by Thoemmes (2012). PSM is a statistical tool used in order to make a causal inference in studies in which randomized sampling is not possible, as it adjusts for the effects of measured confounders (Rosenbaum and Rubin, 1983). PSM is a two-step procedure, estimating the propensity scores (PS) and matching on the basis of these PS. PS were estimated with logistic regression using survey time point [pre- (0) versus peri- (1) pandemic survey] as the dependent variable and selected covariates of loneliness as predictors, which were based on a previous review by Cohen-Mansfield et al. (2016). These covariates included gender, age, marital status, income level, education level, employment status, subjective health, number of people in the household, and level of care allowance and social support. The number of living children was added as a predictor, as social contact with children had emerged as an important coping mechanism under Covid-19 in our survey. 1:1 nearest neighbor matching was performed with a caliper of 0.15, as per scientific standard (Thoemmes, 2012), resulting in a sample of *n* = 888 individuals (444: 444). The matched sample (*n* = 888) was deemed an improvement over the unmatched sample (*n* = 2563) as all variables (with the exception of marital status) were found to be more balanced over the two groups (Figure 1). The overall χ^2^ imbalance test was non-significant (χ^2^ (11) = 3.60, *p* = 0.98), and L1 measure was smaller after matching = 0.97 than before matching = 0.98, indicating that matching had improved overall balance of the variables.

### Measures

#### Loneliness

Loneliness was measured using the German version of the 6-Item De Jong Gierveld loneliness scale, which has been found to be a reliable and valid measure of loneliness (Gierveld and Tilburg, 2006). The German version used in the pre- and peri-pandemic survey was adopted from the German Aging survey (DEAS), a longitudinal survey of the German population aged 40 and over. Six statements were read out to the participant, which the participant then had to rank from very applicable (1) to not applicable (4) to their own experience. Three of the statements were posed as negatives (e.g., “I often feel rejected”) and were therefore reversed. The average score of the loneliness scale (1–4) was used as the overall measure of loneliness with higher scores describing more loneliness.

#### Covariates Used in PSM

Most of the variables mentioned above were survey in a closed answer format using categories or levels. Numbers of living children and people co-residing with the individual were noted and included in the analysis. Age was described using the participants’ year of birth. Social support (based on the F-SozU-6; Kliem et al., 2015) was calculated by averaging the participant’s scores on four items on social support in their everyday life (e.g., “There are multiple people who I like to spend time with”) with lower scores denoting a higher level of social support (1–4).

## Results

The sample (*n* = 888) was made up of elderly (*M* = 73 years, *SD* = 8.17 years, range = 60–99 years), predominantly female (59%) and married (57%) participants (26% widowed, 13% divorced) with at least one living child at the time of survey (91%). Over sixty percent of the sample reported living with at least one other person (54% with one other person). The majority of the sample reported to be in very good or good health (60%). Only a small number of people reported bad (*n* = 68) or very bad (*n* = 11) health. 75% of participants earned between 1000 and 3000€ per month; almost 90% of all surveyed individuals were retired. Representative for the population, a majority of the sample achieved a low level of formal education (60% finished secondary school). Almost 90% of the sample did not qualify for care allowance, suggesting good functional status. Mean social support score was *M* = 1.63 (*SD* = 0.56), suggesting that on average the sample was well supported. The full range of loneliness scores was reported in both groups (very low loneliness to very high loneliness) with 75% of all people reporting low loneliness scores. Less than 3% of the sample reported to have rather high or very high loneliness. Average loneliness was *M* = 1.67 (*SD* = 0.58) suggesting that the sample was not very lonely.

**FIGURE 2:**
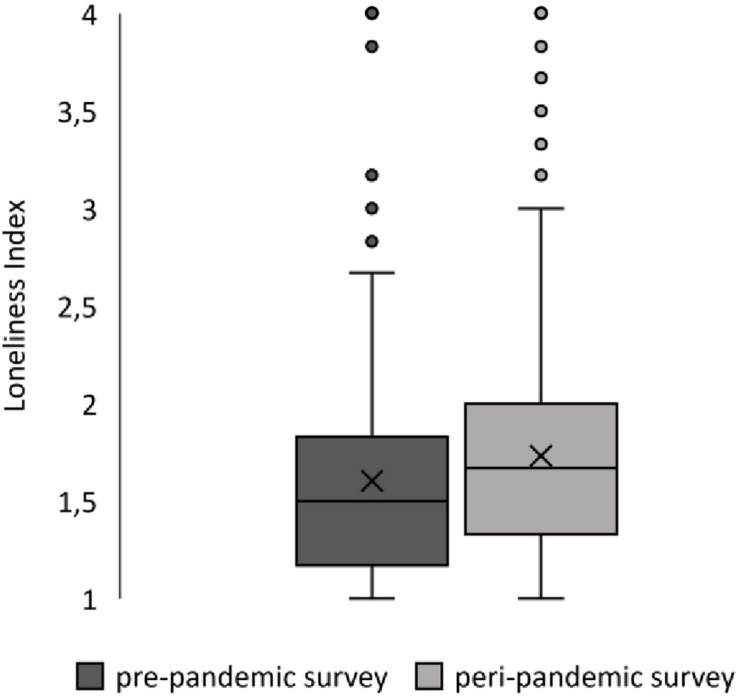
Differences in reported loneliness between pre- and peri-pandemic surveys. Box plots show the distribution and mean value of reported loneliness on pre- and peri-pandemic survey time points. X=mean value, outliers show that lonelier participants were present, if atypical in both surveys.

**FIGURE 3:**
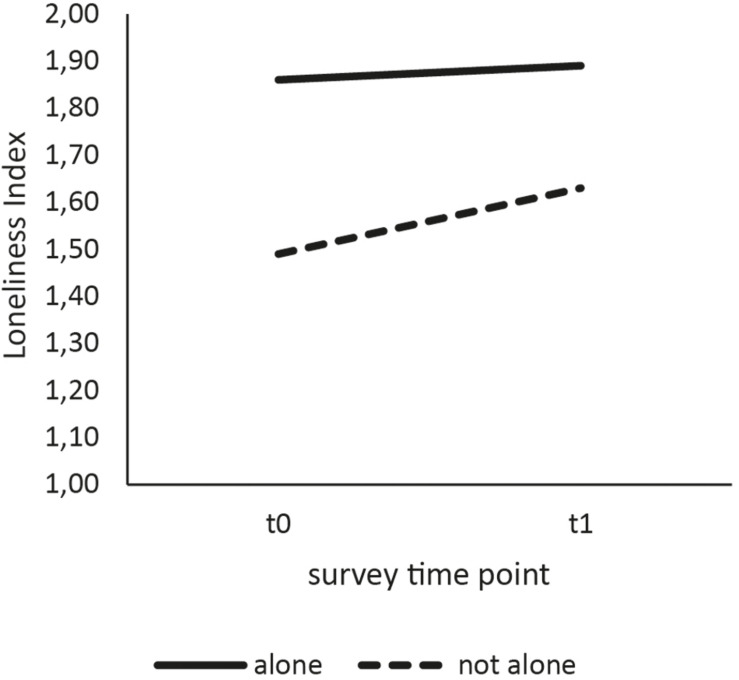
Differences in reported loneliness between time points according to living arrangement.

For comparative analysis, both groups were slightly reduced due to missing values in the loneliness variable (*n*_0_ = 418, *n*_1_ = 435). A difference in loneliness scores could be reported with individuals surveyed prior to the COVID-19 pandemic having a lower average score *M*_0_ = 1.61 (*SD* = 0.55) than individuals surveyed during the COVID-19 pandemic *M*_1_ = 1.73 (*SD* = 0.60). This increase in scores (Figure 2) was interpreted as the peri-pandemic group being slightly more lonely than the pre-pandemic group. This difference was shown to be significant [*t*(851) = −3.17, *p* = 0.002, *d* = 0.22], allowing a generalization of this finding to the population of elderly, community- dwelling people. To explore differences in more detail, subgroups of participants living alone versus with at least one other person were compared on their loneliness scores (Figure 3). Welch ANOVA revealed significant differences between groups [*F*(3, 388.87) = 22.97; *p* < 0.001], which were investigated by using Games–Howell *post hoc* analysis. Participants living with at least one person reported to be less lonely than participants who were living alone at both the pre-pandemic (−0.37, *p* < 0.001) and the peri-pandemic (−0.26, *p* < 0.001) measurement. While participants living with at least one other person reported a significantly higher mean loneliness score during the pandemic as compared to before Covid-19 measures (0.14, *p* = 0.01), participants living alone did not (0.03, *p* = 0.97).

## Discussion

Comparative analysis showed a slight, but significant increase in loneliness among the elderly of Lower Austria during Covid-19 safety measures. Keeping the small effect size in mind, this result could be indicative of a negative trend of distancing measures leading to more loneliness in an elderly population. This negative development of loneliness had been suggested in many scientific and popular communications, but has, to our knowledge, not been analyzed with the exception of two prior studies (Luchetti et al., 2020; van Tilburg et al., 2020). Our findings are in accordance with a Dutch online study (van Tilburg et al., 2020), which showed that elderly community-dwelling citizens reported elevated loneliness during the pandemic and its associated measures. Furthermore, they are also in accordance with the aforementioned American study (Luchetti et al., 2020) which reported a slight increase in loneliness among the group of elderly Americans (65+) after the introduction of social distancing measures, which then remained stable over time. As our study only uses the data of one peri-pandemic survey, we cannot remark upon changes over time during Covid-19 measures but rather compare data from before and during Covid-19 crisis and its associated measures. Due to the vast differences between Austria, the Netherlands, and the United States of America in the handling of Covid-19 (promptness of action, enforcement), we cannot assume absolute similar circumstances between these studies but can report analogical results for a European sample of elderly community-based citizens in a country with a particularly quick reaction to the viral spread.

Concerning the assessment of differences between subgroups, our study found an increase in loneliness among participants who were living with at least one other person but not in participants living alone. Participants who were living alone reported higher loneliness than those in a multi-person household at both time points, which is in accordance with previous scientific work (Victor et al., 2005; Perissinotto et al., 2012). However, persons living alone did not show significantly higher loneliness during the pandemic as compared to before the pandemic. This perplexing finding may indicate a difference in vulnerability to the social changes of the pandemic. It is plausible that people living alone did not experience the safety measures equally as restrictive, as persons living with at least one another person, possibly, as they were more used to being alone. Being alone has also been shown to be related but not equal to loneliness, with previous work pointing out that a person may feel lonely even when surrounded by others (Hawkley and Cacioppo, 2010; Perissinotto et al., 2012). It may also be the case that persons in single-person households were more self-effective and therefore more resilient during safety measures or (virtual) social contacts have increasingly concentrated on people living alone. This cannot be verified with the available data, but it seem logical that people considered at risk of loneliness were more frequently contacted by friends and relatives. Therefore, it is necessary for future studies to examine whether addressing persons living alone as a vulnerable group during the crisis has led to a buffer effect in this group.

Seeing as Austria had a relatively controlled spread of Covid-19 and therefore only a short period of strict safety measures including isolation and social distancing, loneliness may be even more affected in other countries that had a less positive progression. In the context of the varied handling of Covid-19 across nations, this would be an important area for further research especially in relation to possible vulnerability/protective factors (living arrangements, social contact). Additional research is particularly important, as loneliness relates to negative health and well-being outcomes (Cohen-Mansfield et al., 2016; Valtorta et al., 2016), making increased loneliness a general issue. Although previous research concerning loneliness in younger age brackets has been mixed (Losada-Baltar et al., 2020; Luchetti et al., 2020; Pearman et al., 2020), this study shows a negative trend in the substantial group of older adults, which must be considered and possibly prevented in future handling of the ongoing pandemic, as even small differences in loneliness have been shown to affect health and well-being outcomes (Hawkley and Cacioppo, 2010). A slight rise in loneliness during the time of Covid-19 safety measures may therefore have dire consequences in the short and long term. Hence, the reported findings should draw the attention of both scientists and policymakers as they demonstrate a negative development in loneliness among the elderly during this time and should be considered for the ongoing handling of this and future medical crises.

## Data Availability Statement

The datasets presented in this article are not readily available because two datasets are used for statistical analysis of which one sets is not authorized for distribution. Requests to access the datasets should be directed to theresa.heidinger@kl.ac.at.

## Ethics Statement

Ethical review and approval was not required for the study on human participants in accordance with the local legislation and institutional requirements. Written informed consent for participation was not required for this study in accordance with the national legislation and the institutional requirements. However, consent was implied via completion of the survey.

## Author Contributions

TH was the primary author of this manuscript. Analysis and finalization were done in collaboration with LR. Both authors contributed to the article and approved the submitted version.

## Conflict of Interest

The authors declare that the research was conducted in the absence of any commercial or financial relationships that could be construed as a potential conflict of interest.
